# Supplementary material for: SARS due to COVID-19: Predictors of death and profile of adult patients in the state of Rio de Janeiro, 2020
Source: PLoS One. 2022 Nov 10;17(11):e0277338. doi: 10.1371/journal.pone.0277338 (PMC9648756; doi:10.1371/journal.pone.0277338)
Supplement: S2 File — (DOCX) [file pone.0277338.s002.docx]

**Variables Dictionary**

| **Order** | **Variable name** | **Categories** | **Description** |
| --- | --- | --- | --- |
| 1 | obito_evolucao_sim | 0 – no  1 – yes | 1 - Death due to COVID-19 SARS  0 - Cure (non-death) |
| 2 | FX_ETARIA | 18-49 years  50-64 years  65-74 years  75 years and more | Age group |
| 3 | CS_SEXO | M – male  F – female | Sex |
| 4 | CS_RACA | Branco - white  Não branco - non white | Race/skin colour |
| 5 | IDADE2 | Not applicable | Age (in years) |
| 6 | SATURACAO | 1 – yes  2 - no | Saturation less than 95% |
| 7 | DIARREIA | 1 – yes  2 – no | Diarrhoea |
| 8 | VOMITO | 1 – yes  2 – no | Vomiting |
| 9 | GARGANTA | 1 – yes  2 – no | Odynophagia |
| 10 | DISPNEIA | 1 – yes  2 – no | Dyspnoea |
| 11 | DESC_RESP | 1 – yes  2 – no | Respiratory distress |
| 12 | FEBRE | 1 – yes  2 – no | Fever |
| 13 | TOSSE | 1 – yes  2 – no | Cough |
| 14 | FATOR_RISC | 1 – yes  2 – no | At least one risk factor/comorbidity |
| 15 | CS_GESTANT | 0 – no  1 – yes | Pregnancy |
| 16 | PUERPERA_2 | 0 – no  1 – yes | Puerperal period |
| 17 | CARDIOPATI_2 | 0 – no  1 – yes | Cardiovascular disease |
| 18 | RENAL_2 | 0 – no  1 – yes | Chronic kidney disease |
| 19 | NEUROLOGIC_2 | 0 – no  1 – yes | Chronic neurological disease |
| 20 | PNEUMOPATI_2 | 0 – no  1 – yes | Chronic lung disease |
| 21 | OBESIDADE_2 | 0 – no  1 – yes | Obesity |
| 22 | ASMA_2 | 0 – no  1 – yes | Asthma |
| 23 | IMUNODEPRE_2 | 0 – no  1 – yes | Immunosuppression |
| 24 | DIABETES_2 | 0 – no  1 – yes | Diabetes mellitus |
| 25 | HEMATOLOGI_2 | 0 – no  1 – yes | Haematological disease |
| 26 | HEPATICA_2 | 0 – no  1 – yes | Chronic liver disease |
| 27 | EXAME.DE.IMAGEM.II | Atípico COVID-19 – Atypical COVID-19  Normal - Normal  Típico COVID-19 - Typical COVID-19 | Imaging examination (X-ray and chest tomography) |
| 28 | ANTIVIRAL | 1 – yes  2 – no | Use of antiviral |
| 29 | HOSPITAL | 1 – yes  2 – no | Hospitalization |
| 30 | UTI | 1 – yes  2 – no | Use of intensive care |
| 31 | SUPORT_VEN | 1 – invasive  2 - noninvasive  3 – no support | Use of ventilatory support |
| 32 | CRITERIO | Clínico - Clinical  Clínico-epidemio – Clinical-epidemiological  Laboratorial - Laboratory | COVID-19 confirmation criteria |
